# Genomic landscape of gliosarcoma: distinguishing features and targetable alterations

**DOI:** 10.1038/s41598-021-97454-6

**Published:** 2021-09-09

**Authors:** Mark M. Zaki, Leila A. Mashouf, Eleanor Woodward, Pinky Langat, Saksham Gupta, Ian F. Dunn, Patrick Y. Wen, Brian V. Nahed, Wenya Linda Bi

**Affiliations:** 1 Center for Skull Base and Pituitary Surgery, Department of Neurosurgery, Brigham and Women’s Hospital, 60 Fenwood Road, Boston, MA 02115 USA; 2 Department of Neurosurgery, University of Oklahoma Health Sciences Center, Oklahoma City, OK USA; 3 Center for NeuroOncology, Dana-Farber Cancer Institute, Harvard Medical School, Boston, MA USA; 4 Department of Neurosurgery, Massachusetts General Hospital, Harvard Medical School, Boston, MA USA; 5 Department of Neurosurgery, University of Michigan, Ann Arbor, MI USA

**Keywords:** Cancer, Neuroscience, Biomarkers, Oncology

## Abstract

Gliosarcoma is an aggressive brain tumor with histologic features of glioblastoma (GBM) and soft tissue sarcoma. Despite its poor prognosis, its rarity has precluded analysis of its underlying biology. We used a multi-center database to characterize the genomic landscape of gliosarcoma. Sequencing data was obtained from 35 gliosarcoma patients from Genomics Evidence Neoplasia Information Exchange (GENIE) 5.0, a database curated by the American Association of Cancer Research (AACR). We analyzed genomic alterations in gliosarcomas and compared them to GBM (n = 1,449) and soft tissue sarcoma (n = 1,042). 30 samples were included (37% female, median age 59 [IQR: 49–64]). Nineteen common genes were identified in gliosarcoma, defined as those altered in > 5% of samples, including *TERT* Promoter (92%), *PTEN* (66%), and *TP53* (60%). Of the 19 common genes in gliosarcoma, 6 were also common in both GBM and soft tissue sarcoma, 4 in GBM alone, 0 in soft tissue sarcoma alone, and 9 were more distinct to gliosarcoma. Of these, *BRAF* harbored an OncoKB level 1 designation, indicating its status as a predictive biomarker of response to an FDA-approved drug in certain cancers. *EGFR*, *CDKN2A*, *NF1*, and *PTEN* harbored level 4 designations in solid tumors, indicating biological evidence of these biomarkers predicting a drug-response. Gliosarcoma contains molecular features that overlap GBM and soft tissue sarcoma, as well as its own distinct genomic signatures. This may play a role in disease classification and inclusion criteria for clinical trials. Gliosarcoma mutations with potential therapeutic indications include *BRAF, EGFR, CDKN2A, NF1*, and *PTEN*.

## Introduction

Gliosarcoma is a rare tumor histologically characterized by both glial and sarcomatous features^1^, classified by the World Health Organization (WHO) as a grade IV *IDH* wild-type variant of glioblastoma (GBM) with a prevalence of approximately 2% within all adult GBM^2,3^. Despite aggressive treatment consisting of surgical resection, radiation, and chemotherapy, survival for gliosarcoma remains poor with a median survival of 9 months compared to a median 15-month survival for other forms of GBM^1^. It is essential to accurately distinguish gliosarcoma from GBM in order to better inform patient prognosis with this distinct malignancy, as well as to allow for focused translational and clinical investigation into therapies that will improve outcomes for gliosarcoma specifically.

Gliosarcoma presents a diagnostic challenge due to the lack of clear guidelines for clinicians and dedicated scientific focus on gliosarcoma as a separate entity from GBM; it is not unusual for clinical trials to group GBM and gliosarcoma together in patient inclusion criteria without subsequent stratification in analysis^4–7^. Gliosarcoma can be divided into primary and secondary gliosarcoma, with secondary subtypes thought of arising from previously treated GBM^8^. Primary gliosarcoma is historically perceived as a rare subtype of malignant glioma with similarly poor prognosis to glioblastoma. The rarity of this disease, however, has made it difficult to unequivocally define distinguishing features.

In this study, we use a genomic database from an international consortium to characterize molecular alterations in gliosarcoma, GBM, and soft tissue sarcomas to better delineate shared and distinguishing features between these tumors. To help guide therapeutic strategies, we also highlight genetic alterations in gliosarcoma that are currently targeted with existing therapies for other indications.

## Methods

### Sample identification

We identified 37 samples from 35 patients annotated as gliosarcoma from the American Association of Cancer Research (AACR) Genomics Evidence Neoplasia Information Exchange (GENIE) database^9^. Patient age in the cohort is captured as "< 18″ or the exact age when > 18. For two patients with two tumor samples each, the sample with a later date of collection was excluded, as well as two others labeled as local recurrence or metastasis. Three primary gliosarcoma cases which shared a patient identification number with GBM tumor samples were also excluded. In total, 30 tumor samples from 30 distinct patients remained for analysis, derived from Memorial Sloan Kettering Cancer Center (MSK, n = 12), MD Anderson (MDA, n = 7), Dana-Farber Cancer Institute (DFCI, n = 8), and Johns Hopkins University (JHU, n = 3) (Supplementary Table 1).

We compared these thirty gliosarcoma samples to the genomic profile of GBM (n = 1449) and soft tissue sarcoma (n = 1042) in GENIE. As with the gliosarcoma cohort, we excluded samples which were not primary tumors and limited our analysis to one tumor per patient, selecting the earlier specimen in cases of multiple samples belonging to a single patient.

### Sequence variant calling

Institutions contributing samples to the present study (MSK, MDA, JHU, and DFCI) reported minimum depths of sequencing coverage of 750 ×, 250 ×, 500 ×, and 200 ×, respectively; with an average tumor sequencing coverage depth of 200 × to 4000 × and average depth of variant coverage of 10 × to 500 × (AACR GENIE Data Guide, v7.0-public). Individual variant-calling parameters were employed by each institution: MSK sample alterations were detected from matched tumor-normal sequence data, with sequence mutations reported for > 5% allele frequency for novel variants or > 2% allele frequency for recurrent hotspots. MDA sequence variant identification also incorporated germline variant subtraction, with variant filters of > 5% allele frequency, minimum variant coverage > 25, and absence of the variant in paired germline DNA. JHU specimens had multiple variant filters including a total read depth filter ≥ 100, variant allele coverage ≥ 10, variant allele frequency for substitutions ≥ 0.05, and variants seen in greater than 20% of a set of non-neoplastic control tissues were excluded. DFCI variant identification similarly used a panel of historical normal samples to filter putative germline variants.

### Genomic analysis

An average of 247 genes were assayed per sample, with some degree of variability in the total and specific genes assayed at each institution (Supplementary Table 1). To account for this variability in determining the prevalence of genomic alterations in gliosarcoma, we excluded genes assayed in fewer than one-third of all sequenced samples for both mutations (n = 10) and copy number alterations (n = 7). To focus on potentially clinically meaningful changes, we considered only mutations, fusions, and high-level (2 or more copies) amplifications or losses, as defined by the GENIE data dictionary^9^. Genes that were altered in greater than 5% of assayed samples for each tumor type, with a minimum a genetic alteration in > 2 samples, were considered common alterations for the purpose of this study.

### Targetable mutations

We partitioned observed genomic alterations by the OncoKB classification system: (1) an FDA-recognized biomarker predictive of response to an FDA-approved drug for a specific indication; (2) standard care biomarker recommended by an oncology expert panel predictive of response to an FDA-approved drug for a specific indication; (3) clinical evidence supports the biomarker being predictive of response to an investigational drug in a specific indication or an FDA-approved drug in another indication; and (4) biological evidence supports the biomarker being predictive of response to a drug^10^.

### Statistical analysis

Fisher’s Exact Test was used to compare the frequency of altered genes between gliosarcoma, GBM, and soft tissue sarcoma. We then performed a Benjaminin-Hochberg (False Discovery Rate) correction for multiple hypothesis, with a q-value cutoff of 0.1. All analyses were performed using the R programming language, version 3.6.0^11^.

## Results

**Figure 1:**
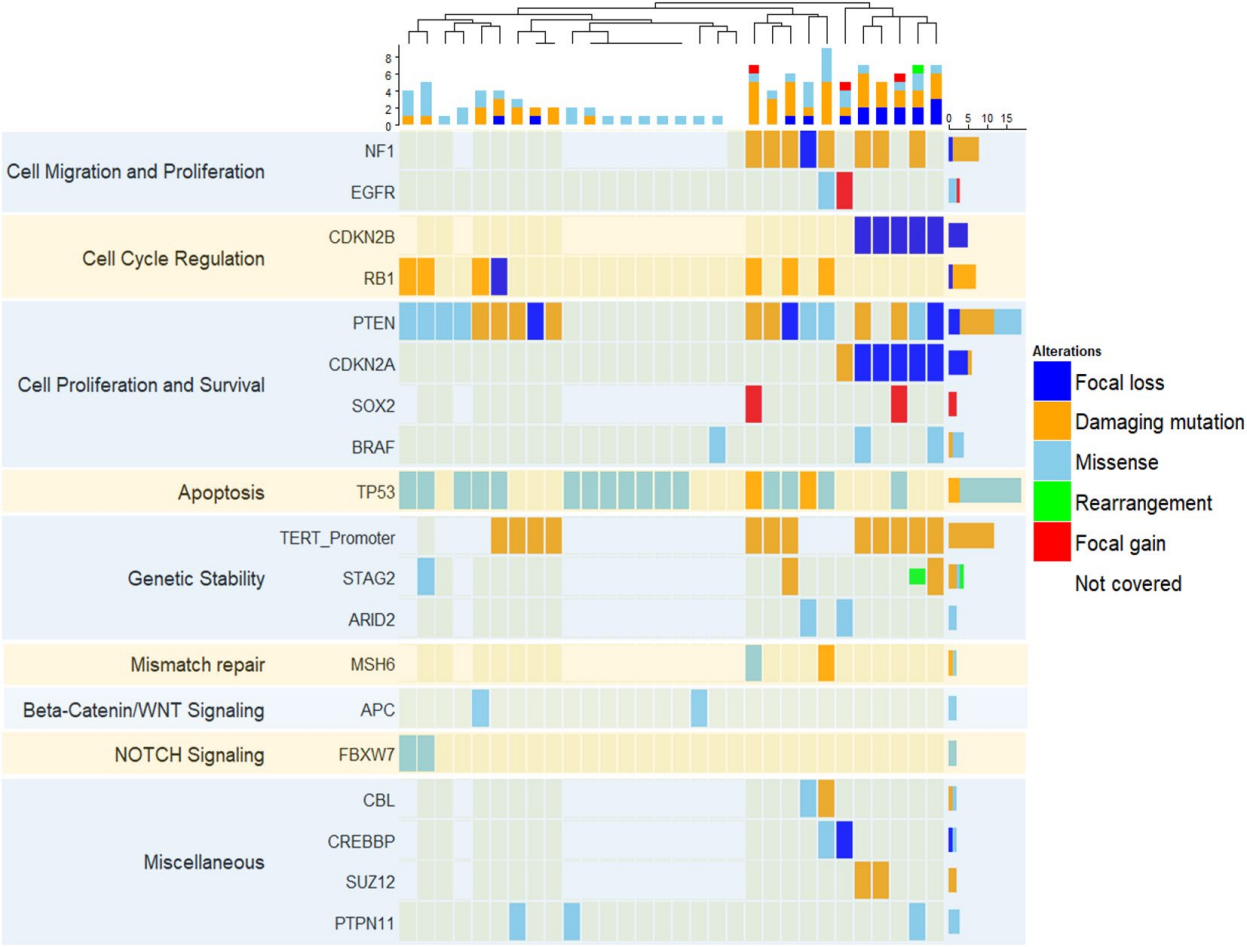
Recurrent genetic alterations in gliosarcoma. Summary of major alterations in 19 most frequently altered genes and pathways in gliosarcoma. Labels on left represent major biological pathways altered by each group of genes, and genes are ordered within each group in order of decreasing incidence. By functional categorization and descending order of mutation frequency: Cell Migration and Proliferation: *NF1* (41%), *EGFR* (12%), Cell Cycle Regulation: *CDKN2B* (28%), *RB1* (26%), *ANKRD11* (11%), Cell Proliferation and Survival: PTEN (66%), *CDKN2A* (31%), *SOX2* (11%), *BRAF* (10%), Apoptosis: *TP53* (60%), Genetic Stability: TERT Promoter (92%), STAG2 (22%), *ARID2* (11%), *Mismatch repair: MSH6* (11%), and Miscellaneous: *CBL* (11%), *CREBBP* (11%), *SUZ12* (11%), *PTPN11* (10%). Order of samples determined by hierarchical clustering. Bar plots above and to the right represent number of alterations per sample and per gene, respectively.

We analyzed 30 patients with gliosarcoma, with median age of 59 years (range: 18–79 years) and a slight predilection for male patients (63%) (Table 1). We identified 19 genes commonly altered genes in gliosarcoma. The most frequently altered were *TERT* Promoter (92%), *PTEN* (66%), *TP53* (60%), and *NF1* (41%), (Fig. 1). Fourteen of 19 recurrently altered genes were associated with mutations, while three (*CDKN2A*, *CDKN2B*, and *SOX2*) harbored copy number alterations, and two (*EGFR* and *CREBBP*) contained both mutations and copy number alterations with nearly equal frequency (Supplementary Table 2). Twenty-nine of 30 samples harbored an alteration in one or more of the common 19 genes (Fig. 1). These genes were associated with multiple recognized cellular pathways including cellular migration, proliferation, survival, cell cycle regulation, apoptosis, and genetic stability^12^.

**Table 1:** Patient Demographics for GENIE Gliosarcoma Samples (n = 30).

	Median	Interquartile range	Range
Age	59	49–64	< 18–79

	Male	Female	Ratio
Gender	19	11	1.7:1

	White	Black	Unknown
Race	25	1	4

### Potentially targetable alterations

We assessed the potential clinical impact of observed mutations by scoring them based on the four categories of targetability defined by OncoKB, a precision oncology database collating data on the treatment implications of specific cancer gene alterations^10^. Of the 19 common genes in gliosarcoma, five (*BRAF*, *EGFR*, *CDKN2A*, *NF1*, and *PTEN*) were indicated as potentially targetable genes present in the OncoKB database (Table 2). Of these, *BRAF* harbored a level 1 alteration for several non-CNS cancers, indicating its status as FDA-recognized predictive biomarker of response to an FDA-approved drug; notably, this FDA indication does not include gliosarcoma. The approved drugs target the well-established *BRAF*^*V600E*^ mutation, which represented two of the four *BRAF* mutations in our cohort, for a prevalence of 7% (n = 2 of 30). Another four targetable alterations (*EGFR*, *CDKN2A*, *NF1*, and *PTEN*) were classified as level 4 for solid tumors, indicating the existence of compelling biological evidence for predictive value of response to an existing drug.

**Table 2:** Targetable alterations in gliosarcoma.

Gene	Alteration type	Protein alterations	Alteration frequency (%)	Combined alteration frequency (all alteration types) (%)	Unique to gliosarcoma?	OncoKB Tier
TERT promoter	Mut	*Noncoding mutations occurring at hotspots C228T and C250T*	92	92	FALSE	N/A
PTEN	Mut/CNA	C71Y, G230*, G36R, L325P, N184Efs*6, N48K, R130*, R130Q, R173C, R233*, S229*, V166Sfs*14, V175M, W274*, X55_splice, X268_splice, Deep deletion	50/17	67	FALSE	Level 4 for oncogenic mutations in all solid tumors
TP53	Mut	C135F, C238Y, D281G, H179Y, H193R, I255N, K132R, L111P, P80Lfs*43, R175H, R248Q, R248W, R273C, R282W, R342*, S241F, T125M, V272M, V73Wfs*50, Y205H	60	60	FALSE	N/A
NF1	Mut/CNA	E1264*, I1679_Y1680del, P1847Qfs*16, Q2589*, R1534*, R2637*, Y2285Tfs*5, Deep deletion	35/6	41	FALSE	Level 4 for oncogenic mutations in all solid tumors
CDKN2A	CNA	Deep deletion	31	31	FALSE	Level 4 for oncogenic mutations in all solid tumors
CDKN2B	CNA	Deep deletion	28	28	FALSE	N/A
RB1	Mut/CNA	H733Ffs*13, M484Vfs*8, R467*, S149*, S567*, S576Rfs*34, Deep deletion	20/6	26	FALSE	N/A
STAG2	Mut	G935Vfs*2, K906Nfs*11, M318R	17	17	FALSE	N/A
EGFR	Mut/CNA	A289V, R222C, Amplification	7/5	12	FALSE	Level 4 for amplification and A289V in gliomas
ARID2	Mut	I124T, T1180K	11	11	TRUE	N/A
CBL	Mut	R420L, R718*	11	11	TRUE	N/A
MSH6	Mut	L1244dup, T1133A	11	11	TRUE	N/A
SUZ12	Mut	G42Afs*30, T596Nfs*6	11	11	TRUE	N/A
SOX2	CNA	Amplification	11	11	TRUE	N/A
CREBBP	Mut/CNA	A1603T, Deep deletion	6/6	11	TRUE	N/A
BRAF	Mut	G32_A33dup, G466E, V600E	10	10	TRUE	Level 1 for V600E in certain non-glioma cancers (e.g. melanoma, colorectal, thyroid, and lung cancers.)
PTPN11	Mut	G60R, N308D, S502L	10	10	FALSE	N/A
FBXW7	Mut	R465H, R465C	7	7	TRUE	N/A
APC	Mut	A735V, R876Q	7	7	TRUE	N/A

The top 19 genes in Gliosarcoma (those altered in at least 10% of cases) and their respective level of targetability per OncoKB.

*indicates truncating mutation due to early stop codon.

### Comparison of gliosarcoma with related tumors

**Figure 2:**
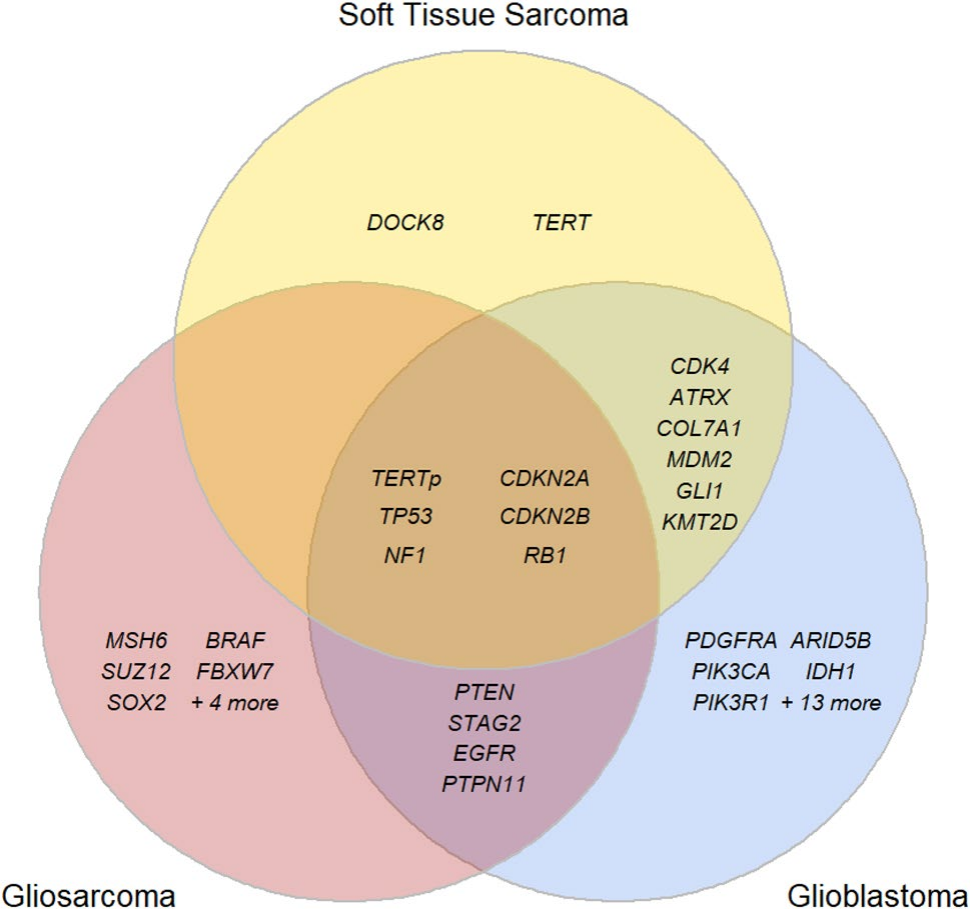
Genetic alteration comparison. Venn diagram of representative commonly altered genes in gliosarcoma, glioblastoma (GBM), and soft tissue sarcoma.

To help delineate unique and shared features of gliosarcoma compared to other tumors, we next analyzed genes that were altered in greater than 5% of GBM (n = 1449) and soft tissue sarcoma (n = 1042) samples in GENIE (Supplementary Figure 1). Thirty-four common genes were identified in GBM and 14 were identified in soft tissue sarcoma. Among all the samples, 6 were considered common along tumor types. Gliosarcoma shared 4 genes with GBM alone, none with soft tissue sarcoma alone, and the remaining nine common genes in gliosarcoma were unique to gliosarcoma amongst the 5% threshold for each respective tumor type (Fig. 2, Supplementary Table 4).

**Figure 3:**
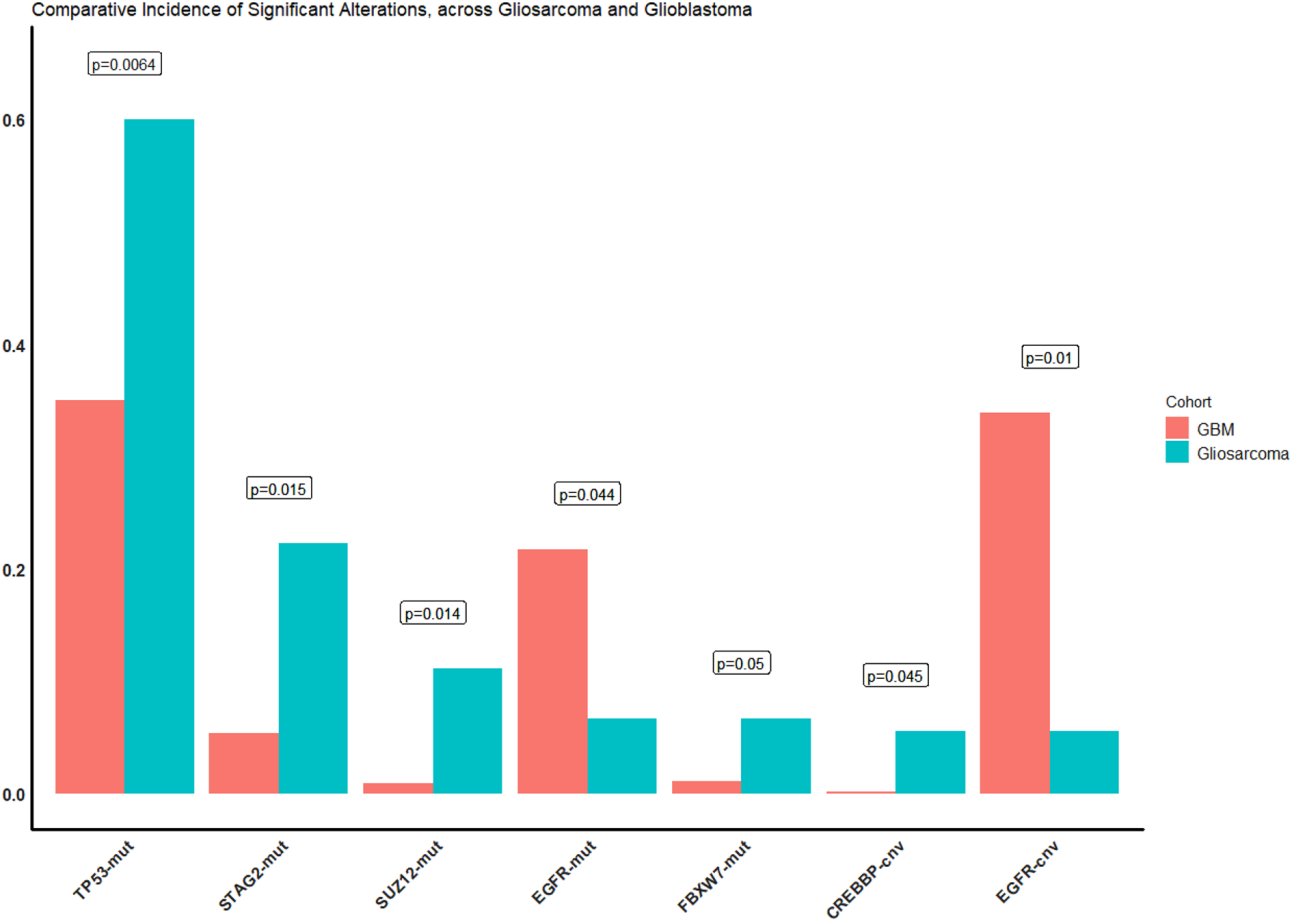
Comparative incidence of common alterations between GBM and gliosarcoma. Incidence of alterations which significantly differed between gliosarcoma and GBM (Fisher’s Exact Test *p* < 0.05), although no differences retained significance after multiple hypothesis correction.

When comparing the alteration frequencies these genes, *EGFR*, *TP53*, *SUZ12*, and *CREBBP* were significantly different between gliosarcoma and GBM, (Fisher’s Test, *p* < 0.05), but none maintained significance when corrected for multiple hypothesis testing (Fig. 3). Alteration frequencies of multiple genes including *TP53*, *TERT* Promoter and *PTEN*, were significantly different between gliosarcoma and soft tissue sarcoma (Fisher’s Test, *p* < 0.05; FDR correction, q < 0.01) (Supplementary Table 3).

## Discussion

With a growing understanding of the molecular alterations driving gliomas, scientific effort towards tumor-specific, personalized therapies has been robust^13^. Gliosarcomas are rare entities, however, and there is sparse data on their molecular profile. In comparison to GBM, gliosarcomas exhibit a higher propensity for extracranial metastasis and differing response to therapies when compared to GBM^14–16^. In this study, we build upon the emerging literature on the genomic signatures of gliosarcoma.

Although the WHO classification places gliosarcoma as a variant within the greater category of glioblastoma, the distinct mutational landscapes between the two may provide evidence that the common practice of grouping GBM and gliosarcoma patients in clinical trials may not be appropriate. Based on our findings and support in the literature of significant genomic differences between gliosarcoma and glioblastoma, it would be of interest to stratify responses to treatment between GBM and gliosarcoma in order to better understand clinical responses based on tumor type, although the small number of gliosarcoma patients may make this difficult. Additionally, our findings demonstrating no specific mutations shared exclusively between gliosarcoma and soft tissue sarcoma suggest that association between these two entities may be vestigial. The lack of genetic overlap linking soft tissue sarcoma and gliosarcoma reported here should be considered in future studies and taxonomy.

In our study, we observed *BRAF* to be altered in 10% of gliosarcomas, compared to 3% of GBMs. *BRAF*^*V600E*^ in particular is a level 1 OncoKB target for many cancers. While evidence has been limited, one case study of two patients diagnosed with GBM harboring *BRAF*^*V600E*^ mutations demonstrated tumor regression and control after treatment with a dual BRAF-MEK inhibitor, though treatment resistance developed, limiting survival to 7 and 7.5 months respectively^17^. Another case of a pediatric GBM patient with a *BRAF*^*V600E*^ mutation demonstrated complete clinical regression 6 months post-treatment with *BRAF* inhibitor therapy^18^. Other studies have shown that targeting *BRAF*^*V600E*^ shows promising results in a variety of gliomas^19–21^. *BRAF* may thus be a therapeutic avenue for a subset of gliosarcomas as well.

In our study, we found *EGFR* altered in 12% of gliosarcoma samples. Other genomic analyses of gliosarcoma have found frequencies of *EGFR* amplification of 4% in a study of 22 samples and 74% *EGFR* gain in another study of 18 samples with one sample expressing *EGFR* amplification^22,23^. Differences in epidermal growth factor receptor (EGFR) mutation type and frequency contrast gliosarcoma from GBM. EGFR is thought to be a key oncogenic driver in GBM, amplified in 35–45% of IDH wildtype glioblastomas^24^. Investigation of EGFR-targeted therapies for GBM has been robust and diverse, including anti-EGFR antibody-loaded nanoparticles, anti-EGFRvIII CAR-T therapy, antibody drug conjugates like depatuxizumab mafodotin, and clinical trials investigating monoclonal antibodies or tyrosine kinase inhibitors against EGFR like erlotinib and lapatinib^25–29^. According to OncoKB, lapatinib has a level 4 indication for glioma, suggesting biological evidence for potential success as a targeted therapy. In gliosarcoma, no independent trial with EGFR targeted agents has been published to our knowledge. In a comprehensive whole-genome copy number analysis of gliosarcoma, a study found *EGFR* amplification was uncommon, but found frequent gains of chromosome 7, which contains the *EGFR* locus, among other genes including *CDK6*, *PDGF*-A, and c-*Met*^22^. It is unclear if the EGFR pathway is indirectly activated in gliosarcoma through other mutations. Therapies targeting *EGFR* mutations are unlikely to be important treatment options in gliosarcoma due to low frequency of genetic alterations.

Interestingly, one study found *TP53* mutations in gliosarcoma to be correlated with worse prognosis, treatment resistance, and epithelial to mesenchymal transition of the sarcomatous cell population, making it a prospective marker for prognostic categorization^1^. Other mutations seen in high frequency in this particular analysis of gliosarcoma include *TERT* promoter, *CDK2NB, RB1, and STAG2*.

Other genes, elicited in our analysis, showing potential opportunities for investigation include *PTEN, NF1,* and *CDKN2A* belonging generally to the Ras/PI3K/AKT pathway. Overall, these mutations remain of unclear clinical and prognostic relevance, though present an interesting avenue for further development as markers of prognosis or tumor-specific therapy.

In our study, *PTEN* had an alteration frequency of 67% among gliosarcoma samples. *PTEN* alterations have previously been detected in 26% of HGG in TCGA data, and identified in 45% (9/20) of previously investigated gliosarcomas^30,31^. Loss of *PTEN* function through deletion, mutation or down regulation has been found to potentially enrich sensitivity to small molecule inhibitors of *PI3Kβ* such as AZD8186 and GSK2636771, as studied in cell line panels and early clinical trials of patients with *PTEN*-deficient advanced solid tumors including GBM, respectively^32,33^.

We further observed alterations in *NF1* in 41% of gliosarcomas via damaging mutations and copy number losses. Loss of NF1 function enhances *RAS* activity, inducing *RAS/RAF/MEK/ERK* pathway activation. Single-agent *MEK* inhibitors (PD0325901 and AZD6244) have been shown to be effective against a subset of *NF1*–deficient GBM cells dependent on *RAF/MEK/ERK* signaling^34^.

We found *CDKN2A* had an alteration frequency of 31% among the gliosarcoma samples, specifically representing copy number losses and an inactivating mutation. *CDNK2A* loss is common in 35–60% of GBMs and independently associated with worse overall and progression-free survival in both molecularly and histologically defined *IDH*-wildtype GBM^31,35–37^ In a microarray study of gliosarcomas, homozygous loss of *CDKN2A* was previously detected in 14 of 18 gliosarcoma specimens studied^38^. Given its significance as a poor prognostic marker in GBM, further investigation into the implications of *CDKN2A* loss in gliosarcoma is warranted.

Our findings demonstrate a noteworthy distinction between gliosarcoma and GBM, as well as illuminate the potential for robust genomic and histologic analysis of this unique tumor type for prognostication and therapeutics. Histologically, gliosarcomas demonstrate intratumoral heterogeneity, notably of a biphasic composition with both glial-like and mesenchymal/sarcomatous cell populations^23,39^. Several studies suggest that these populations may be monoclonal in origin given their similar expression of early known glial mutations including p53^23,39^. A greater fraction of the sarcomatous component in comparison to a predominantly glial component has also been associated with survival benefit (71 vs 63 weeks) in small case series^40^. Intratumoral heterogeneity between these distinct cellular subpopulations in gliosarcoma harbingers acquired resistance to a single targeted therapy, as suggested in other glial tumors^41^. This provides another opportunity for further study to characterize the potential heterogeneous composition of this tumor.

With a better understanding of the unique genomic landscape of gliosarcoma, scientific focus may be given to developing gliosarcoma-specific therapeutics. Among the most frequently altered genes found in gliosarcoma, *BRAF, EGFR, PTEN, NF1*, and *CDKN2A* are potentially targetable according to OncoKB. Limitations of this study include the finite data set collected and presented within the GENIE database used for our analysis. Specifically, no clinical data was available to correlate genomic alterations with clinical outcomes in this study, nor were further details on histologic characterization of these tumors available. Additionally, institutions reporting to the GENIE database utilize differing parameters in their genetic assays, including in the average depth of coverage and quality parameter thresholds for variant reporting. This heterogeneity within the database itself limits an ideal level of standardization in our determination of significant variants.

## Conclusion

While gliosarcoma is categorized by the WHO as a variant of glioblastoma and often grouped with GBM in clinical trials, this study suggests that gliosarcoma has a genomic landscape distinct from GBM and soft tissue sarcoma. These differences should influence disease classification as well as guide targeted therapy for this aggressive tumor.

## Supplementary Information

Supplementary Information.
