# Supplementary material for: Genomic landscape of gliosarcoma: distinguishing features and targetable alterations
Source: Sci Rep. 2021 Sep 9;11:18009. doi: 10.1038/s41598-021-97454-6 (PMC8429571; doi:10.1038/s41598-021-97454-6)
Supplement: Supplementary file 1 — Supplementary Information. [file 41598_2021_97454_MOESM1_ESM.docx]

**Title:** Genomic Landscape of Gliosarcoma: Distinguishing Features and Targetable Alterations

**Short title:** Genomic Landscape of Gliosarcoma

**Authors** Mark M. Zaki, BS, BA^1^; Eleanor Woodward, BS^1^; Leila Mashouf, BS^1^; Saksham Gupta, MD^1^; Ian F. Dunn, MD^2^; Patrick Wen, MD^3^; Brian V. Nahed, MD, MSc^3^; Wenya Linda Bi, MD, PhD^1^

^1^Center for Skull Base and Pituitary Surgery, Department of Neurosurgery, Brigham and Women’s Hospital, Harvard Medical School, Boston, MA

^2^Department of Neurosurgery, University of Oklahoma Health Sciences Center, Oklahoma City, OK

^3^Center for NeuroOncology, Dana-Farber Cancer Institute, Harvard Medical School, Boston, MA

^4^Department of Neurosurgery, Massachusetts General Hospital, Harvard Medical School, Boston, MA

**Conflict of interests**: none for all authors

**Disclosures**: none for all authors

**Funding**: none for all authors

**Post-Publication Corresponding Author**:

Wenya Linda Bi, MD, PhD

Center for Skull Base and Pituitary Surgery

Department of Neurosurgery

Brigham and Women’s Hospital
60 Fenwood Road

Boston, MA 02115

Phone : 617-525-8319
Fax : 617-713-3050
E-mail : [wbi@bwh.harvard.edu](mailto:wbi@bwh.harvard.edu)

**Supplementary Figure 1.**

1. Glioblastoma Comut Plot


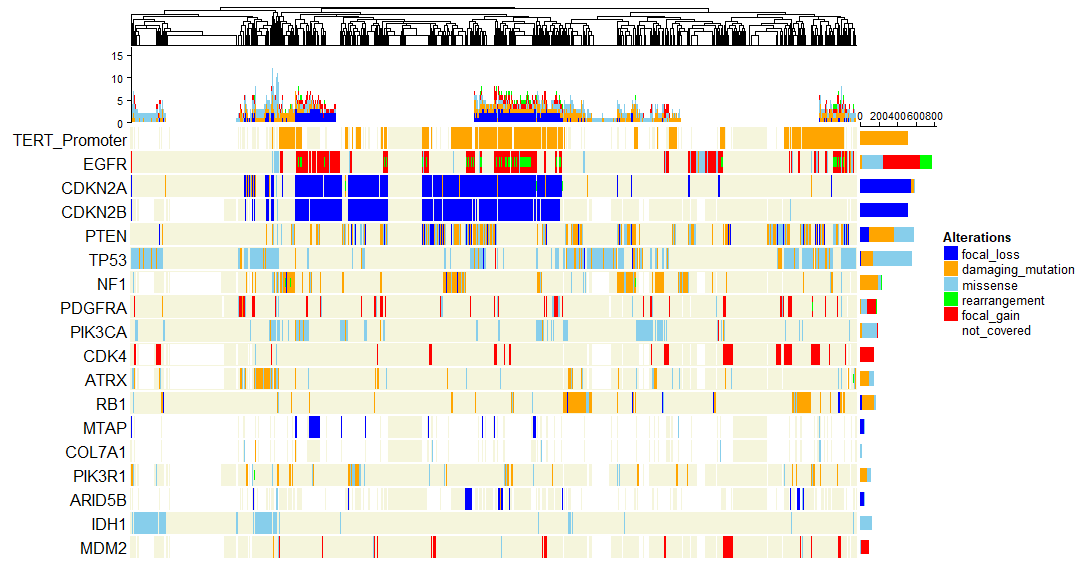


1. Soft Tissue Sarcoma Comut Plot


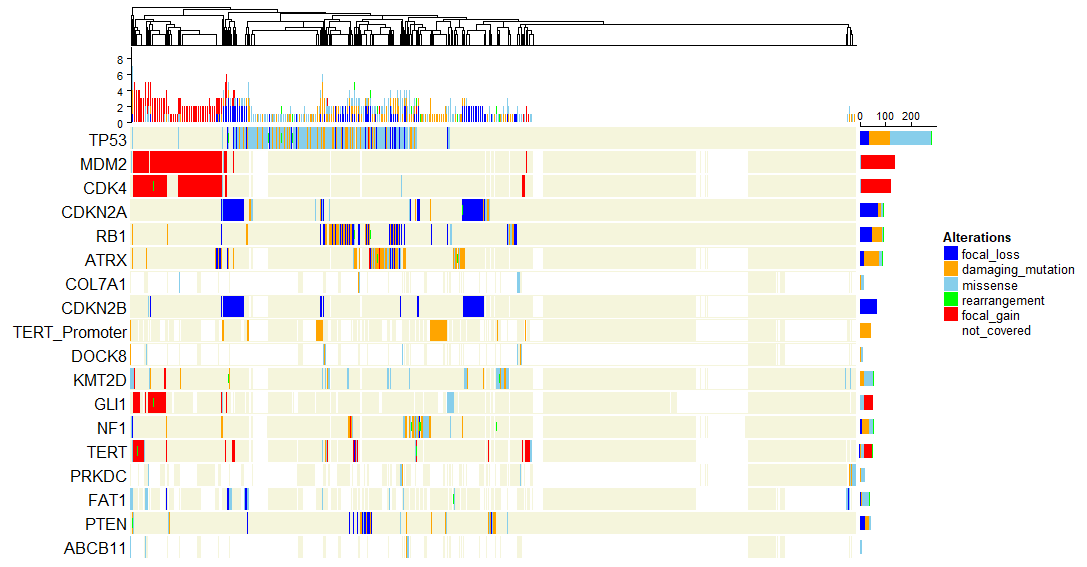


**Supplementary Table 1.** Contributing GENIE Institutions

| **Institution** | **Panel** | **No. of Genes Assayed** | **No. of Cases** |
| --- | --- | --- | --- |
| MSK | MSK-IMPACT341 | 341 | 4 |
|  | MSK-IMPACT410 | 410 | 4 |
|  | MSK-IMPACT468 | 468 | 4 |
| MDA | MDA-46-V1 | 46 | 2 |
|  | MDA-50-V1 | 50 | 5 |
| JHU | JHU-50GP-V2 | 40 | 3 |
| DFCI | DFCI-ONCOPANEL-1 | 275 | 1 |
|  | DFCI-ONCOPANEL-2 | 300 | 4 |
|  | DFCI-ONCOPANEL-3 | 447 | 1 |
|  | GRCC-MOSC4 | 82 | 2 |

**Supplementary Table 2.** Total Alterations per Sample

| Sample | Total  No. of Alterations | No. of Mutations | No. of Copy Number Alterations (CNAs) (All) | | No. of CNAs (High-level only) | | No. of Structural Variants | Sequencing Assay |
| --- | --- | --- | --- | --- | --- | --- | --- | --- |
|  |  |  | + | - | + | - |  |  |
| GENIE-DFCI-002987-63 | 39 | 2 | 18 | 19 | 0 | 0 | 0 | DFCI-ONCOPANEL-1 |
| GENIE-DFCI-007570-4277 | 110 | 4 | 17 | 89 | 0 | 0 | 0 | DFCI-ONCOPANEL-2 |
| GENIE-DFCI-008626-7049 | 101 | 5 | 8 | 88 | 2 | 1 | 0 | DFCI-ONCOPANEL-2 |
| GENIE-DFCI-010523-10381 | 139 | 6 | 130 | 3 | 2 | 1 | 0 | DFCI-ONCOPANEL-2 |
| GENIE-DFCI-037378-73845 | 132 | 29 | 86 | 17 | 0 | 0 | 0 | DFCI-ONCOPANEL-2 |
| GENIE-DFCI-037074-88660 | 88 | 10 | 22 | 56 | 0 | 5 | 0 | DFCI-ONCOPANEL-3 |
| GENIE-GRCC-z221npmm-sample-a | 5 | 5 | 0 | 0 | 0 | 0 | 0 | GRCC-MOSC4 |
| GENIE-GRCC-e2ddkv4y-sample-a | 0 | 0 | 0 | 0 | 0 | 0 | 0 | GRCC-MOSC4 |
| GENIE-JHU-01841-02250 | 2 | 2 | 0 | 0 | 0 | 0 | 0 | JHU-50GP-V2 |
| GENIE-JHU-03996-04667 | 1 | 1 | 0 | 0 | 0 | 0 | 0 | JHU-50GP-V2 |
| GENIE-JHU-04154-04848 | 1 | 1 | 0 | 0 | 0 | 0 | 0 | JHU-50GP-V2 |
| GENIE-MDA-2285-4440 | 2 | 2 | 0 | 0 | 0 | 0 | 0 | MDA-46-V1 |
| GENIE-MDA-2287-4442 | 1 | 1 | 0 | 0 | 0 | 0 | 0 | MDA-46-V1 |
| GENIE-MDA-3132-5662 | 1 | 1 | 0 | 0 | 0 | 0 | 0 | MDA-50-V1 |
| GENIE-MDA-4953-15967 | 2 | 2 | 0 | 0 | 0 | 0 | 0 | MDA-50-V1 |
| GENIE-MDA-5743-16652 | 1 | 1 | 0 | 0 | 0 | 0 | 0 | MDA-50-V1 |
| GENIE-MDA-5984-16909 | 1 | 1 | 0 | 0 | 0 | 0 | 0 | MDA-50-V1 |
| GENIE-MDA-6678-17919 | 2 | 2 | 0 | 0 | 0 | 0 | 0 | MDA-50-V1 |
| GENIE-MSK-P-0000653-T01-IM3 | 8 | 8 | 0 | 0 | 0 | 0 | 0 | MSK-IMPACT341 |
| GENIE-MSK-P-0001049-T01-IM3 | 2 | 2 | 0 | 0 | 2 | 1 | 0 | MSK-IMPACT341 |
| GENIE-MSK-P-0001053-T01-IM3 | 6 | 6 | 0 | 0 | 0 | 0 | 0 | MSK-IMPACT341 |
| GENIE-MSK-P-0002138-T01-IM3 | 8 | 7 | 0 | 1 | 1 | 0 | 0 | MSK-IMPACT341 |
| GENIE-MSK-P-0006848-T01-IM5 | 6 | 6 | 0 | 0 | 0 | 0 | 0 | MSK-IMPACT410 |
| GENIE-MSK-P-0008380-T01-IM5 | 3 | 3 | 0 | 0 | 0 | 1 | 0 | MSK-IMPACT410 |
| GENIE-MSK-P-0009649-T01-IM5 | 6 | 6 | 0 | 0 | 0 | 3 | 0 | MSK-IMPACT410 |
| GENIE-MSK-P-0011026-T01-IM5 | 4 | 4 | 0 | 0 | 2 | 2 | 0 | MSK-IMPACT410 |
| GENIE-MSK-P-0015347-T01-IM6 | 6 | 6 | 0 | 0 | 0 | 2 | 0 | MSK-IMPACT468 |
| GENIE-MSK-P-0016784-T01-IM6 | 2 | 2 | 0 | 0 | 0 | 0 | 0 | MSK-IMPACT468 |
| GENIE-MSK-P-0021478-T01-IM6 | 5 | 4 | 0 | 0 | 0 | 2 | 1 | MSK-IMPACT468 |
| GENIE-MSK-P-0021832-T01-IM6 | 6 | 6 | 0 | 0 | 0 | 1 | 0 | MSK-IMPACT468 |

**Supplementary Table 3.** Comparison of Gliosarcoma alterations frequencies with GBM and Soft Tissue Sarcoma (STS)

| **Gene** | **Alteration Type** | **Gliosarcoma samples altered (n)** | **Samples Assayed (n)** | **Gliosarcoma Alteration Frequency (%)** | **GBM Alteration Frequency (%)** | **STS Alteration Frequency (%)** | **Gliosarcoma vs. GBM p-value** | **Gliosarcoma vs. STS p-value** | **Gliosarcoma vs. GBM q-value (FDR corr.)** | **Gliosarcoma vs. STS q-value (FDR corr.)** | **Gliosarcoma frequency closer to GBM than STS?** |
| --- | --- | --- | --- | --- | --- | --- | --- | --- | --- | --- | --- |
| TERT Promoter* | mut | 12 | 13 | 92.3% | 71.7% | 6.6% | 1.25E-01 | 3.43E-13 | 2.99E-01 | 6.35E-12 | TRUE |
| TP53 | mut | 18 | 30 | 60.0% | 35.0% | 22.3% | 6.41E-03 | 1.34E-05 | 1.40E-01 | 1.65E-04 | TRUE |
| PTEN | mut | 15 | 30 | 50.0% | 32.9% | 2.4% | 7.58E-02 | 2.26E-15 | 2.54E-01 | 8.37E-14 | TRUE |
| NF1 | mut | 7 | 20 | 35.0% | 18.2% | 5.0% | 7.56E-02 | 5.34E-05 | 2.54E-01 | 3.95E-04 | TRUE |
| CDKN2A | cna | 5 | 18 | 27.8% | 46.8% | 7.7% | 1.52E-01 | 1.13E-02 | 3.20E-01 | 3.48E-02 | FALSE |
| CDKN2B | cna | 5 | 18 | 27.8% | 44.2% | 7.3% | 2.31E-01 | 8.98E-03 | 4.07E-01 | 3.02E-02 | FALSE |
| STAG2 | mut | 4 | 18 | 22.2% | 5.3% | 0.7% | 1.51E-02 | 1.85E-05 | 1.40E-01 | 1.71E-04 | TRUE |
| RB1 | mut | 6 | 30 | 20.0% | 10.7% | 4.5% | 1.29E-01 | 2.60E-03 | 2.99E-01 | 1.20E-02 | TRUE |
| PTEN | cna | 3 | 18 | 16.7% | 8.2% | 2.2% | 1.85E-01 | 8.25E-03 | 3.42E-01 | 3.02E-02 | TRUE |
| ARID2 | mut | 2 | 18 | 11.1% | 3.0% | 2.0% | 1.09E-01 | 5.75E-02 | 2.88E-01 | 1.18E-01 | TRUE |
| CBL | mut | 2 | 18 | 11.1% | 2.0% | 1.0% | 5.59E-02 | 1.68E-02 | 2.54E-01 | 4.59E-02 | TRUE |
| MSH6 | mut | 2 | 18 | 11.1% | 2.5% | 1.4% | 8.10E-02 | 3.07E-02 | 2.54E-01 | 7.10E-02 | TRUE |
| SUZ12 | mut | 2 | 18 | 11.1% | 0.9% | 1.0% | 1.37E-02 | 1.74E-02 | 1.40E-01 | 4.59E-02 | TRUE |
| SOX2 | cna | 2 | 18 | 11.1% | 3.9% | 0.1% | 1.57E-01 | 1.03E-03 | 3.20E-01 | 6.35E-03 | TRUE |
| BRAF | mut | 3 | 30 | 10.0% | 3.5% | 1.2% | 8.93E-02 | 8.67E-03 | 2.54E-01 | 3.02E-02 | TRUE |
| PTPN11 | mut | 3 | 30 | 10.0% | 5.9% | 0.7% | 4.18E-01 | 2.08E-03 | 6.73E-01 | 1.10E-02 | TRUE |
| APC | mut | 2 | 30 | 6.7% | 2.3% | 3.3% | 1.64E-01 | 2.67E-01 | 3.20E-01 | 4.49E-01 | TRUE |
| EGFR | mut | 2 | 30 | 6.7% | 21.7% | 1.2% | 4.42E-02 | 6.34E-02 | 2.54E-01 | 1.24E-01 | FALSE |
| FBXW7 | mut | 2 | 30 | 6.7% | 1.1% | 1.2% | 4.98E-02 | 5.59E-02 | 2.54E-01 | 1.18E-01 | TRUE |
| CREBBP | mut | 1 | 18 | 5.6% | 4.3% | 3.1% | 5.52E-01 | 4.41E-01 | 8.52E-01 | 7.10E-01 | TRUE |
| CREBBP | cna | 1 | 18 | 5.6% | 0.2% | 0.0% | 4.49E-02 | 1.92E-02 | 2.54E-01 | 4.73E-02 | TRUE |
| EGFR | cna | 1 | 18 | 5.6% | 33.9% | 0.3% | 1.01E-02 | 7.46E-02 | 1.40E-01 | 1.38E-01 | FALSE |
| NF1 | cna | 1 | 18 | 5.6% | 0.4% | 0.9% | 8.79E-02 | 1.61E-01 | 2.54E-01 | 2.83E-01 | TRUE |
| RB1 | cna | 1 | 18 | 5.6% | 1.5% | 5.2% | 2.42E-01 | 1.00E+00 | 4.07E-01 | 1.00E+00 | FALSE |
| CDKN2A | mut | 1 | 30 | 3.3% | 3.0% | 2.1% | 6.08E-01 | 4.83E-01 | 9.00E-01 | 7.45E-01 | TRUE |
| CDKN2B | mut | 0 | 18 | 0.0% | 0.4% | 0.1% | 1.00E+00 | 1.00E+00 | 1.00E+00 | 1.00E+00 | FALSE |
| SOX2 | mut | 0 | 18 | 0.0% | 0.3% | 0.6% | 1.00E+00 | 1.00E+00 | 1.00E+00 | 1.00E+00 | TRUE |
| APC | cna | 0 | 18 | 0.0% | 0.0% | 0.1% | 1.00E+00 | 1.00E+00 | 1.00E+00 | 1.00E+00 | FALSE |
| ARID2 | cna | 0 | 18 | 0.0% | 0.4% | 0.1% | 1.00E+00 | 1.00E+00 | 1.00E+00 | 1.00E+00 | FALSE |
| BRAF | cna | 0 | 18 | 0.0% | 0.5% | 0.0% | 1.00E+00 | 1.00E+00 | 1.00E+00 | 1.00E+00 | FALSE |
| CBL | cna | 0 | 18 | 0.0% | 0.0% | 0.4% | 1.00E+00 | 1.00E+00 | 1.00E+00 | 1.00E+00 | FALSE |
| FBXW7 | cna | 0 | 18 | 0.0% | 0.1% | 0.3% | 1.00E+00 | 1.00E+00 | 1.00E+00 | 1.00E+00 | TRUE |
| MSH6 | cna | 0 | 18 | 0.0% | 0.1% | 0.0% | 1.00E+00 | 1.00E+00 | 1.00E+00 | 1.00E+00 | FALSE |
| PTPN11 | cna | 0 | 18 | 0.0% | 0.1% | 0.4% | 1.00E+00 | 1.00E+00 | 1.00E+00 | 1.00E+00 | TRUE |
| STAG2 | cna | 0 | 18 | 0.0% | 0.3% | 0.1% | 1.00E+00 | 1.00E+00 | 1.00E+00 | 1.00E+00 | FALSE |
| SUZ12 | cna | 0 | 18 | 0.0% | 0.1% | 0.5% | 1.00E+00 | 1.00E+00 | 1.00E+00 | 1.00E+00 | TRUE |
| TP53 | cna | 0 | 18 | 0.0% | 1.0% | 4.1% | 1.00E+00 | 1.00E+00 | 1.00E+00 | 1.00E+00 | TRUE |

Abbreviations: mutation (mut); copy number alteration (cna).

*TERT Promoter region not specifically annotated for CNAs

Yellow highlight indicates p < .05

Red highlight indicates “false”

**Supplementary Table 4**. Mutation and CNA frequency in genes altered in greater than 5% of samples in GBM and Soft Tissue Sarcoma (STS).

|  |  |  |  |  |  |  |  |
| --- | --- | --- | --- | --- | --- | --- | --- |
| GBM |  |  |  |  |  |  |  |
| Gene | No. of samples mutated | No. of samples assayed (mutations) | Mutation Frequency (%) | No. of samples with CNA | No. of samples assayed (CNA) | CNA Frequency (%) | Total Combined Alteration Frequency (%) |
| TERT Promoter | 521 | 727 | 71.7% | NA | NA | 0.0% | 71.7% |
| EGFR | 241 | 1449 | 16.6% | 396 | 1168 | 33.9% | 50.5% |
| CDKN2A | 40 | 1449 | 2.8% | 547 | 1168 | 46.8% | 49.6% |
| CDKN2B | 5 | 1181 | 0.4% | 516 | 1168 | 44.2% | 44.6% |
| PTEN | 477 | 1449 | 32.9% | 96 | 1168 | 8.2% | 41.1% |
| TP53 | 507 | 1449 | 35.0% | 12 | 1168 | 1.0% | 36.0% |
| NF1 | 212 | 1196 | 17.7% | 5 | 1168 | 0.4% | 18.2% |
| PDGFRA | 74 | 1436 | 5.2% | 98 | 1168 | 8.4% | 13.5% |
| PIK3CA | 184 | 1449 | 12.7% | 9 | 1168 | 0.8% | 13.5% |
| CDK4 | 5 | 1181 | 0.4% | 149 | 1168 | 12.8% | 13.2% |
| ATRX | 142 | 1181 | 12.0% | 3 | 1168 | 0.3% | 12.3% |
| RB1 | 153 | 1449 | 10.6% | 17 | 1168 | 1.5% | 12.0% |
| MTAP | 5 | 176 | 2.8% | 49 | 592 | 8.3% | 11.1% |
| COL7A1 | 19 | 176 | 10.8% | 0 | 592 | 0.0% | 10.8% |
| PIK3R1 | 115 | 1196 | 9.6% | 1 | 1168 | 0.1% | 9.7% |
| ARID5B | 9 | 551 | 1.6% | 42 | 551 | 7.6% | 9.3% |
| IDH1 | 134 | 1449 | 9.2% | 0 | 1168 | 0.0% | 9.2% |
| MDM2 | 10 | 1181 | 0.8% | 89 | 1168 | 7.6% | 8.5% |
| PIK3C2B | 17 | 617 | 2.8% | 32 | 617 | 5.2% | 7.9% |
| KIT | 28 | 1449 | 1.9% | 65 | 1168 | 5.6% | 7.5% |
| PRKDC | 47 | 630 | 7.5% | 0 | 617 | 0.0% | 7.5% |
| SETD2 | 81 | 1181 | 6.9% | 1 | 1168 | 0.1% | 6.9% |
| KDR | 36 | 1449 | 2.5% | 50 | 1168 | 4.3% | 6.8% |
| GLI1 | 25 | 1103 | 2.3% | 52 | 1168 | 4.5% | 6.7% |
| FANCM | 13 | 201 | 6.5% | 0 | 592 | 0.0% | 6.5% |
| KMT2D | 73 | 1181 | 6.2% | 2 | 1143 | 0.2% | 6.4% |
| USP28 | 11 | 176 | 6.3% | 0 | 592 | 0.0% | 6.3% |
| MDM4 | 9 | 1181 | 0.8% | 64 | 1168 | 5.5% | 6.2% |
| PTPN11 | 85 | 1449 | 5.9% | 1 | 1168 | 0.1% | 6.0% |
| TEK | 5 | 243 | 2.1% | 21 | 551 | 3.8% | 5.9% |
| STAG2 | 63 | 1179 | 5.3% | 3 | 1143 | 0.3% | 5.6% |
| CDKN2C | 21 | 1170 | 1.8% | 42 | 1168 | 3.6% | 5.4% |
| FAT1 | 34 | 750 | 4.5% | 7 | 1168 | 0.6% | 5.1% |
| KAT6B | 9 | 178 | 5.1% | 0 | 592 | 0.0% | 5.1% |
|  |  |  |  |  |  |  |  |
| STS |  |  |  |  |  |  |  |
| Gene | No. of samples mutated | No. of samples assayed (mutations) | Mutation Frequency (%) | No. of samples with CNA | No. of samples assayed (CNA) | CNA Frequency (%) | Total Combined Alteration Frequency (%) |
| TP53 | 229 | 1042 | 22.0% | 38 | 921 | 4.1% | 26.1% |
| MDM2 | 3 | 933 | 0.3% | 134 | 921 | 14.5% | 14.9% |
| CDK4 | 4 | 933 | 0.4% | 118 | 921 | 12.8% | 13.2% |
| CDKN2A | 21 | 1042 | 2.0% | 71 | 921 | 7.7% | 9.7% |
| RB1 | 46 | 1042 | 4.4% | 48 | 921 | 5.2% | 9.6% |
| ATRX | 69 | 933 | 7.4% | 19 | 921 | 2.1% | 9.5% |
| COL7A1 | 14 | 160 | 8.8% | 0 | 406 | 0.0% | 8.8% |
| CDKN2B | 1 | 933 | 0.1% | 67 | 921 | 7.3% | 7.4% |
| TERT Promoter | 44 | 671 | 6.6% | 0 | 0 | 0.0% | 6.6% |
| DOCK8 | 10 | 160 | 6.3% | 1 | 406 | 0.2% | 6.5% |
| KMT2D | 49 | 933 | 5.3% | 3 | 917 | 0.3% | 5.6% |
| GLI1 | 16 | 859 | 1.9% | 34 | 921 | 3.7% | 5.6% |
| NF1 | 43 | 939 | 4.6% | 8 | 921 | 0.9% | 5.4% |
| TERT | 15 | 917 | 1.6% | 34 | 917 | 3.7% | 5.3% |
